# Preliminary Insights into the Association Between Room Transfer and Delirium Symptoms After Orthopedic Surgery: A Retrospective Case-Control Study

**DOI:** 10.7759/cureus.90978

**Published:** 2025-08-25

**Authors:** Yusuke Nitta, Yuri Nakai, Reiko Hashimoto, Hisao Nakai

**Affiliations:** 1 Department of Neuropsychiatry, Kanazawa Medical University, Kahoku, JPN; 2 Faculty of Nursing, University of Kochi, Kochi, JPN

**Keywords:** case control, delta program, orthopedic surgery, postoperative delirium, room transfer

## Abstract

Background: Because of population aging in recent decades, the number of patients at risk of postoperative delirium has increased. Delirium can lead to adverse events such as falls, prolonged hospital stays, and increased mortality, making postoperative delirium prevention a critical issue.

Aim: This study was to identify environmental factors associated with delirium symptoms, which are specifically screened for in STEP 2 of the multidisciplinary Delirium Team Approach (DELTA) Program after orthopedic surgery under general anesthesia.

Methods: This was a retrospective, single-center study using a medical record review. Of the 72 target patients, 36 patients with confirmed STEP 2 delirium symptoms (defined by attention disturbance, altered levels of consciousness, acute onset/fluctuation, and sleep-wake rhythm) within the DELTA Program formed the case group, and the remaining 36 formed the control group. The control group was matched according to age and sex. We also examined other factors, including the number of room transfers, to link the methods to the objective of studying environmental factors. The number of days from surgery to the development of STEP 2 symptoms was determined for the case group, and data for the same number of days were examined for the control group.

Results: The mean age (standard deviation) of the case group was 75.9 (9.5) years, and of the control group was 75.1 (10.0) years. The mean number of days from admission date to the date of surgery was 5.4 (5.4) days for the case group and 4.6 (3.6) days for the control group. Binary logistic regression analysis showed that the factors associated with the presence of STEP 2 symptoms after controlling for the number of days from admission to surgery were the preoperative room changes (odds ratio (OR) 8.61, 95% confidence interval (CI) 1.47-50.42) and the postoperative room changes. (OR 4.10, 95% CI 1.34-12.45).

Conclusion: This study suggests a potential association between room transfers and the development of STEP 2 delirium symptoms after orthopedic surgery under general anesthesia. In wards managed by the DELTA Program, avoiding room changes both pre- and post surgery may help reduce the incidence of postoperative delirium. However, as this was a retrospective study, we couldn't adjust for all confounding factors such as surgery type or blood loss. Therefore, these findings are not definitive, and further detailed investigations are necessary to confirm this relationship.

## Introduction

Postoperative delirium is defined as an acute and transient disturbance of consciousness and cognition, with an onset within hours to days following surgery, which is precipitated by the physiological and psychological stressors of the surgical experience [1]. Delirium can be caused by a variety of factors, including severe illness, drugs, and hospitalization [2].

Specifically, triggering factors, such as acute events and stressors, including surgery, anesthesia, infection, and changes or withdrawal of medications, as well as patient vulnerability factors, such as advanced age, a history of dementia, multiple comorbidities, and impaired hearing or vision, have been identified as predisposing patients to delirium [3, 4]. The contributors to delirium are complex and multifactorial, and both patient vulnerability and triggering factors need to be considered. Once delirium develops, there is a risk of treatment interruption, adverse events such as falls, and functional decline, which can prolong hospital stay and increase mortality [5].

The number of older adults aged 65 and above is increasing around the world. Global life expectancy has increased by more than eight years since 1990, reaching 72.6 years in 2019, and is expected to reach 77.1 years by 2050 [6]. Because of population aging in recent decades, the number of patients at risk of delirium in acute care wards has increased [7]. When older adults develop delirium, the onset of dementia may be accelerated in patients with pre-existing cognitive impairment. Furthermore, delirium in older adults increases the risk of death in the hospital [8]. To prevent delirium in older people, interprofessional cooperation and intervention are needed to address risk factors for delirium [9]. In Japan, the number of hospitalized older patients is increasing owing to the birthrate decline and population aging [10]. If a hospitalized patient develops delirium, treatment for the main disease is interrupted, functional decline owing to complications occurs, and hospital stay is lengthened [11]. In response to the increase in delirium in hospitals in Japan, the National Cancer Center has developed the Delirium Team Approach (DELTA) Program, which offers non-pharmacological treatment for delirium. The DELTA Program comprises six components that address delirium prevention and treatment, which are as follows: Multidisciplinary education, 2. Screening, 3. Planning, 4. Prevention, 5. Scheduled evaluation, and 6. Management and treatment [12]. This program provides basic education about delirium to nurses, unifies care, enables information sharing among multiple professions, and provides early detection and treatment through preventive care and regular assessments [13]. The program has demonstrated a reduction in delirium incidence and in problems such as falls and self-extubation of IV drips in cancer patients [14].

Age, impaired cognitive status, sensory impairment, and comorbidities are most frequently cited as major risk factors for postoperative delirium [15]. Several non-pharmacological approaches to avoid treatment interruption owing to postoperative delirium have been recommended, including sleep-wake regulation, orientation strategies, early mobilization, vision and hearing optimization, and nutrition and hydration [16]. Risk factors for postoperative delirium include visual impairment, dementia, and preoperative delirium [17]; furthermore, benzodiazepines have also been identified as a risk factor for delirium [18]. Therefore, interventions to improve the daily rhythm of postoperative patients and the treatment environment are important in preventing postoperative delirium. Research in many fields has demonstrated a relationship between delirium and sensory impairment, which makes it difficult to transmit stimuli from the outside world [19]. Studies of older hospitalized patients have shown that visual and hearing impairments are associated with delirium [20]. One systematic review of studies of adult patients receiving palliative care suggested that sensory impairment may be a risk factor for delirium [21]. Therefore, it is likely that environmental factors within the ward may affect the development of delirium.

There is much evidence that the care environment in intensive care units (ICUs) is associated with delirium. Inadequate daytime light exposure may contribute to abnormalities in circadian rhythmicity [22]. Exposure to natural light from windows is associated with lower rates of delirium in the ICU [23] and is reported to reduce agitation episodes and hallucinations [24]. Improving sound, lighting control, floor plans, and room layout in the ICU could help to create a treatment environment that minimizes stress factors and supports the prevention and management of delirium [25]. Furthermore, there is evidence that the incidence of delirium may be related to the number of times a patient is moved between rooms [26]. Reports on older patients indicate that frequent environmental changes owing to unit or room transfers and lack of consistency of care may contribute to the onset and prolongation of delirium [27]. The risk of delirium may be lower in patients in private rooms than those in multibed rooms [28], and the length of stay in the corridor until an inpatient bed is assigned at the emergency department is associated with the onset of delirium [29]. However, no studies have investigated the relationship between environmental factors and the development of delirium in patients undergoing surgery under general anesthesia.

This study targeted orthopedic surgery patients who had the highest number of consultations with the psychiatric liaison team at a university hospital, working to prevent delirium using the DELTA Program. A study in orthopedic patients reported that delirium was a predictor of functional decline in activities of daily living (ADL) and instrumental activities of daily living (IADL) in orthopedic patients under general anesthesia [30]. Postoperative delirium in elderly patients with femoral neck fractures has been shown to be significantly associated with increased short- and long-term mortality and decreased functional and cognitive recovery [31]. Additionally, active geriatric consultation has been reported to be effective in preventing postoperative delirium in hip fracture patients [32].

The purpose of the study was to identify environmental factors associated with delirium symptoms (STEP 2 symptoms) in the DELTA Program after orthopedic surgery under general anesthesia in the orthopedic ward. The findings may help to inform environmental changes to prevent delirium in wards that have implemented the DELTA Program. Specifically, improving the ward environment by considering factors related to symptoms may contribute to the prevention of postoperative delirium.

## Materials and methods

The DELTA Program

This is a multidisciplinary initial delirium response program that uses non-pharmacological treatment for delirium. The program was developed at the National Cancer Center (Tokyo) in Japan and is run by nurses, doctors, and pharmacists who have received DELTA Program training. The response flow is as follows. Nurses use a dedicated assessment sheet to assess the risk of delirium in STEP 1 upon admission using several delirium risk factors, including age 70 years or older, organic brain disorder, dementia, heavy alcohol consumption, history of delirium, and benzodiazepine usage. In STEP 2, we screen for the following delirium symptoms: attention disturbance, altered levels of consciousness, acute onset or fluctuation of symptoms, and sleep-wake rhythm. Given that environmental changes are major precipitating factors for delirium, the assessment of these symptoms is conducted with particular attention to recent events, such as a room transfer. Patients with any of these STEP 2 conditions are defined as showing STEP 2 symptoms. In other words, it is at this point that the doctor determines whether symptoms of delirium are present. If such symptoms are present, treatment for delirium is provided in STEP 3. These three stages constitute the DELTA Program [12, 14].

Data collection

This was a retrospective, single-center, case-control study based on a medical record review. The study population consisted of patients admitted to the orthopedic ward of Kanazawa Medical University Hospital, Kahoku, Japan, from November 2020 to March 2021 who underwent surgery under general anesthesia; these served as the inclusion criteria. Patients meeting these criteria were consecutively enrolled, as there were no specific exclusion criteria applied during the study period. From this eligible population, all patients who developed STEP 2 symptoms were included using consecutive sampling, forming the case group (n=36). For the control group (n=36), patients without STEP 2 symptoms were selected from the same population using an individual matching technique based on age and sex.

**Figure 1:**
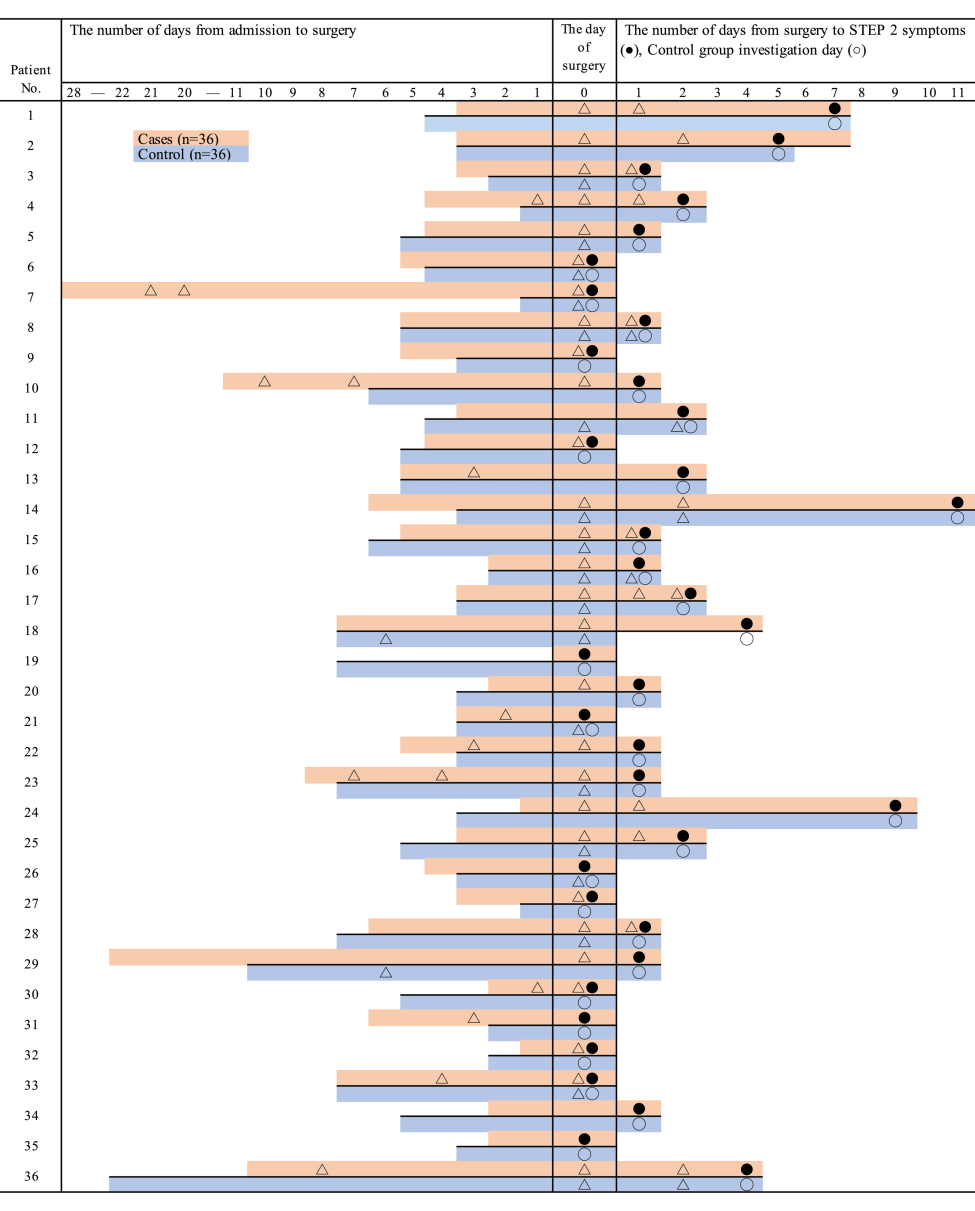
STEP 2 symptoms by number of room changes and treatment environment before and after surgery △ Room change; ● Number of days from surgery to presence of STEP 2 symptoms; ○ Control group investigation day

One of the authors retrospectively collected all data by manually reviewing each patient's electronic medical record (EMR). No specific database or software was used for automated data extraction. The STEP 1 delirium risk factor data were obtained from the dedicated DELTA Program assessment sheet, which is a structured form completed by nurses upon admission and integrated into the EMR. Other variables were extracted from physician and nursing notes and hospital administrative records within the EMR. The observation period for the case group was defined as the time from surgery to the date of STEP 2 symptom onset. An equivalent observation period was established for each matched control patient, starting from the day of surgery. The primary exposure variable was the number of room transfers that occurred during this postoperative period. We also collected retrospective data on the number of days from admission to surgery; the number of room changes before surgery; room location at admission and at the time of STEP 2 symptom onset (categorized as room connected to nurse station, room near nurse station, or room away from nurse station); room type at admission and at symptom onset (single bedroom or multi-bed room); and whether the patient was discharged home. Figure 1 illustrates the data collection timeline for each case-control pair.

Kanazawa Medical University Hospital is one of the largest medical centers in the Hokuriku region, with 39 departments and 817 beds, located in Ishikawa Prefecture, Japan. The hospital has been using the DELTA Program to prevent delirium since 2017, and it was introduced in the orthopedic ward in the same year.

We also collected retrospective data on the number of days from admission to surgery, the room changes before surgery, postoperative room changes, outcomes of room location at admission, room location at STEP 2 symptoms (room connected to nurse station, room near nurse station, room away from nurse station), room type (single bedroom, multibed room) at admission, when it was determined that STEP 2 symptoms were present, and whether or not patients were discharged home.

Data analysis

The analysis population included all patients who met the established inclusion criteria. The mean and standard deviation were calculated for each group for age, number of days from admission date to surgery date, development, number of room changes before surgery, and number of room changes after surgery. For the baseline characteristics, the frequency and percentage of each STEP 1 delirium risk factor were calculated for the case and control groups. To examine the factors that affected postoperative STEP 2 symptoms, we examined the presence or absence of STEP 2 symptoms, sex, age, number of days from admission to surgery, the room changes before and after surgery, and STEP 2 symptoms. We evaluated the relationship between room location at the time of symptoms (room connected to the nurses' station, other hospital room (room near the nurses' station, room away from the nurses' station)), room type (single bedroom, multibed room), and whether or not the patient was discharged home using the chi-square (χ²) test or Fisher’s exact test.

The presence or absence of STEP 2 symptoms was the dependent variable, and the confounding factor was the number of days from admission to surgery. The independent variables were the room changes before and after surgery (1: No, 2: Yes), which had a significance level of <5% in the χ² test or Fisher’s exact test. A binary logistic regression analysis was performed. Each variable was forced into the model after checking for multicollinearity (variance inflation factor ≥10). The significance level was set at 5%. IBM SPSS Statistics software, version 29 (IBM Corporation, Armonk, NY), was used for all statistical analyses.

Ethical considerations

This research was conducted in accordance with the Declaration of Helsinki, 1995 (as revised in Seoul, 2008), and was carried out with the consent of the Kanazawa Medical University medical research ethics review committees at the authors’ universities (No. I144). Given the retrospective nature of the study, the ethics committee waived the requirement for individual informed consent from the participants. To protect patient confidentiality, all data collected from the medical records were fully anonymized before analysis.

## Results

Patients’ background

The background characteristics of the participants are summarized in Table 1. As a result of matching, the case and control groups were identical in sex distribution, with each group comprising 18 males (50.0%) and 18 females (50.0%). There was no significant difference in the mean age between the case group (75.9 ± 9.5 years) and the control group (75.1 ± 10.0 years) (p = 0.614). The mean number of days from admission to surgery also did not differ significantly between the groups (Table 1).

**Table 1:** Patient background and STEP 2 symptoms by delirium risk (n=72) a: Student's t-test; b: Fisher's exact test; c: chi-square (χ^2^) test

			Delirium symptoms (as defined by STEP 2 of the DELTA Program)			
Items	Category	Total	No (n=38)	Yes (n=38)	Test statistic	p value
Background						
Sex	Male	36 (50.0)	18 (50.0)	18 (50.0)	0	1.000 ^c^
	Female	36 (50.0)	18 (50.0)	18 (50.0)		
Age (years), mean±SD		75.5±9.68	75.1±10.0	75.9±9.5	-0.315	0.754 ^a^
Number of days from admission to surgery, mean±SD		5,0±4.60	4.6±3.6	5.4±5.4	-0.69	0.492^ a^
Delirium risk						
Preoperative room changes, n(%)	No	59 (81.9)	34 (57.6)	25 (42.4)	N/A	0.012^ b^
	Yes	13 (18.1)	2 (15.4)	11 (84.6)		
Postoperative room changes, n(%)	No	27 (37.5)	19 (70.4)	8 (29.6)	N/A	0.014 ^b^
	Yes	45 (62.5)	17 (37.8)	28 (62.2)		
Room location at admission, n(%)	Other rooms (room near the nurses' station, room away from the nurses' station)	64 (88.9)	35 (54.7)	29 (45.3)	N/A	0.055^ b^
	Room connected to the nurses' station	8 (11.1)	1 (12.5)	7 (87.5)		
Room location at STEP 2 symptoms, n(%)	Other rooms (room near the nurses' station, room away from the nurses' station)	48 (66.7)	27 (56.3)	21 (43.8)	2.25	0.134^ c^
	Room connected to the nurses' station	24 (33.3)	9 (37.5)	15 (62.5)		
Room types at admission, n(%)	Single bedroom	17 (23.6)	8 (47.1)	9 (52.9)	0.77	0.781^ c^
	Multibed room	55 (76.4)	28 (50.9)	27 (49.1)		
Room types at STEP 2 symptoms, n(%)	Single bedroom	14 (19.4)	8 (57.1)	6 (42.9)	0.35	0.551^ c^
	Multibed room	58 (80.6)	28 (48.3)	30 (51.7)		
Discharged, n(%)	Location other than home	55 (76.4)	30 (54.5)	25 (45.5)	1.92	0.165^ c^
	Home	17 (23.6)	6 (35.3)	11 (64.7)		

STEP 1 delirium risk factors

**Figure 2:**
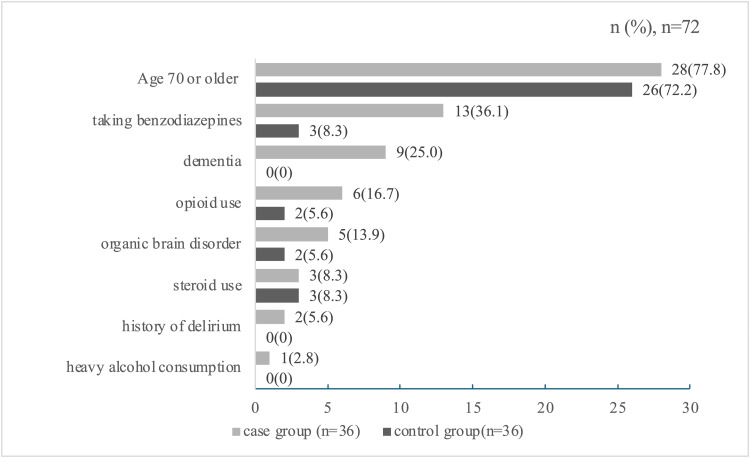
Distribution of delirium risk factors and baseline patient characteristics in case and control groups

In the case group, the most common risk factor was age 70 years or older (n=28, 77.8%). Among the risk factors, the most frequent comorbidity was dementia (n=9, 25.0%), and the most frequently used medication was oral benzodiazepines (n=13, 36.1%). In the control group, the most common risk factor was also age 70 years or older (n=26, 72.2%), while the most frequently used medications were benzodiazepines and steroids (both n=3, 8.3%). Data on these risk factors are shown in Figure 2.

STEP 2 symptoms by the number of room changes and the treatment environment before and after surgery

The mean (standard deviation) number of room changes before surgery in the control group was 0.06 (0.23), and the number of room changes after surgery was 0.64 (0.76). The mean (standard deviation) number of room changes before surgery in the case group was 0.39 (0.65), and the number of room changes after surgery was 1.14 (0.80). Figure 1 shows the progress of STEP 2 symptom determination and room transfer for the control and case groups. The results of the univariate analysis showed that STEP 2 symptoms were associated with the preoperative room changes (p=0.012) and the postoperative room changes (p=0.014) (Table 1).

Treatment environment predictors of STEP 2 symptoms

The results of the binary logistic regression analysis showed that the factors associated with STEP 2 symptoms after adjusting for the effect of the number of days from admission to surgery were the preoperative room changes (odds ratio (OR): 8.61, 95% confidence interval (CI): 1.47-50.42) and the postoperative room changes (OR: 4.10, 95% CI: 1.34-12.45) (Table 2).

**Table 2:** Predictor of STEP 2 symptoms (n=72) The results are from a binary logistic regression analysis. The case and control groups were matched by age and sex. Data for the control group were examined for the same number of days from surgery to STEP 2 symptoms in the case group. OR: odds ratio; 95％ CI: 95% confidence interval

Item	Category	OR	95% CI		P-value
			Lower limit	Upper limit	
Number of days from admission to surgery		0.95	0.84	1.08	0.411
Preoperative room changes	Yes (Ref: No)	8.61	1.47	50.42	0.017
Postoperative room changes	Yes (Ref: No)	4.1	1.34	12.45	0.013

## Discussion

The mean age (standard deviation) of the case group in this study was 75.9 years (9.5), and the control group was 75.1 years (10.0). Regarding STEP 1 of the DELTA Program, that is, delirium risk, age 70 years or older was the most common, with 28 people (77.8%) in the case group and 26 (72.2%) in the control group. A previous study of orthopedic patients found that a randomized trial of postoperative delirium for femoral neck fractures included 79 ± 8 [32]. The mean (standard deviation) age of patients undergoing femoral neck fracture surgery was 78.4 years (9.5 years), and the most frequent complication was delirium, 19% [33]. Furthermore, the mean age (standard deviation) of patients aged 60 years or older who underwent orthopedic surgery and developed postoperative delirium was 74.7 years (7.8 years) [30]. Taking these into account, the subjects of this study may have been typical patients admitted to the orthopedic ward and undergoing surgery under general anesthesia.

This study explored environmental factors potentially linked to STEP 2 delirium symptoms in orthopedic patients undergoing general anesthesia within the DELTA Program. Environmental factors, such as unfamiliar surroundings, sensory deprivation or overstimulation, and sleep disruption, are well-established triggers for delirium, as they can disorient vulnerable patients and disrupt their circadian rhythms [2,4]. Our findings tentatively suggest that preoperative and postoperative room changes might be associated with the appearance of delirium symptoms, even when considering the duration from admission to surgery. This aligns broadly with some prior observations. [34]. While our analysis, controlling for age, sex, and days from surgery to symptom onset, points towards room movement as a possible environmental factor for postoperative delirium in this specific patient group, it's crucial to acknowledge that these results are limited and preliminary. Further robust investigation is essential to solidify these initial observations and deepen our understanding of potential contributors to delirium.

Previous research has indicated that increased patient bed transfers, both within and between wards, can elevate the likelihood of patient falls and wound infections [35]. While bed transfers undoubtedly burden patients, they are often a necessary measure to manage hospital bed shortages and optimize patient flow [36]. We recognize that such transfers can be crucial for efficient hospital management. However, our preliminary findings suggest a potential, though not yet definitive, link between frequent room transfers and postoperative delirium. If confirmed, this could imply that delirium, potentially exacerbated by these transfers, might prolong hospital stays, thereby increasing the burden on medical staff and introducing administrative challenges. For instance, prior studies highlight that nurses spend a significant amount of time, approximately 1,700 hours monthly, on inter-ward patient transfers, which diverts time from direct patient care [37]. Consequently, efforts to prevent postoperative delirium, possibly including minimizing room changes, could also contribute to reducing nursing workload [38]. Ideally, hospital bed turnover rates should be optimized by efficient patient treatment pathways. Given the potential, albeit still unconfirmed, complications of delirium linked to room transfers and the associated workload, our study tentatively supports the idea that bed transfers should be kept to a minimum whenever clinically feasible.

Despite these limitations, our study possesses notable strengths. It is, to our knowledge, one of the first studies in Japan to quantitatively examine the association between room transfers and postoperative delirium within a structured prevention program like the DELTA Program. The primary implication of our preliminary findings is that routine logistical processes, such as patient room changes, may represent a significant and potentially modifiable risk factor for delirium. For future research, prospective studies are essential to confirm this association. Furthermore, interventional studies could be designed to evaluate whether policies aimed at minimizing non-essential room transfers can effectively reduce the incidence of postoperative delirium, thereby contributing to safer hospital environments for vulnerable older surgical patients.

Limitations

This study had several limitations that warrant careful consideration. First, as this was a single-center study, the findings are underpowered, and their generalizability is limited. Second, postoperative delirium is known to be affected by many factors; our study did not adequately address these other potential confounding factors. Furthermore, although data were obtained from medical records, the determination of STEP 2 symptoms may not have been fully consistent owing to variations in the evaluators’ years of experience and skill levels.

Finally, a significant limitation is the observed imbalance in several key baseline delirium risk factors between the groups (Figure 2). Specifically, the case group had a substantially higher prevalence of benzodiazepine use and dementia compared to the control group. Although our primary analysis focused on room changes, we cannot exclude the possibility that these pre-existing risk factors acted as significant confounding variables and contributed to the higher incidence of delirium in the case group. Therefore, a larger multicenter study that employs more advanced statistical methods, such as multivariate logistic regression, is needed to adjust for these multiple risk factors and validate our preliminary observations.

## Conclusions

In this study, we observed a potential association between room transfer before and after surgery and STEP 2 delirium symptoms within the DELTA Program. Based on these preliminary findings, we tentatively suggest that minimizing preoperative and postoperative room transfers might be a useful strategy in wards where delirium prevention measures are implemented through the DELTA Program. This observed, though not yet definitive, association between room transfer and STEP 2 symptoms could offer a potential complement to the existing DELTA Program delirium prevention strategy.
